# Notes and News

**Published:** 1891-12-26

**Authors:** 


### Notes and News.

Collected and Communicated.

The City of London Lying-in-Hospital was instituted in 1750 at Shaftesbury House, Aldersgate Street, and removed to its present site in 1771. It has during the past year attended more patients than in any previous year. The expenditure has exceeded the income by over £500. The Samaritan Fund attached to the hospital affords relief to the poorer patients on leaving. New annual subscriptions are especially wanted for both funds. The Secretary is Mr. R. A. Owthwaite, the Matron Miss S. E. Allen.

The Surgical Aid Society is doing good work. The institution was founded in 1862, since which time it had given no fewer than 147,121 surgical appliances to 101,051 poor persons. During the past year the numbers relieved were 3,470 men, 4,725 women, and 1,246 children, a total of 9,444 individuals, which is the largest number ever relieved in any one year of the society's operations. It is satisfactory to note that the income of last year—£8,478—was larger than that of any previous year, but it still falls far short of what it should be if the society is to be in a position to help all the deserving applicants who came to it for assistance.

Christmas Day commences early in the London Hospital, by the nurses going through the different wards singing carols. The patients much enjoy their dinner, with its roast beef and plum puddings. The evening festivities begin in each ward with a tea of ham, cake, fruit, &c., followed by smoking in the men's wards, and by concerts and entertainments in every ward. The nurses are untiring in their efforts to make this day a memorable one in the lives of the many patients. They have their festivities on the following day. The Christmas season would not be complete in the London Hospital without the well-known entertainments of Punch and Judy shows, combined with a grand Christmas-tree, which will take place on this occasion on January 5. Much taste is shown in the choice decorations in every ward.

Numbers of books have been written on nursery subjects, and numbers of young mothers are still asking the question, "Can you tell me of a good book on the Management of Children." To such enquirers we can answer, "Yes, there really is a publication" par excellence that could fairly be called "The Mother's Guide." It is a periodical, however, that only claims the unpretending title of "Babyhood," and is published in monthly parts, price sixpence, in New York, and at 92, Fleet Street. Casually taking up the number for the present month, we find it so full of useful and comprehensive information, that we almost wonder how enough material can be found to supply its pages with fresh interest from month to month. Yet the task is undoubetly accomplished in this excellent publication, which we unhesitatingly affirm no nursery should be without. The current number, which closes the seventh volume of "Babyhood," commences with some valuable hints on the family medicine chest. This is followed by an admirable paper on the care of infants and delicate children. Then come articles of bathing, dress, diet, and various common nursery troubles. Nor is the mental nurture of the little ones forgotten. We have excellent suggestions on the subject of story telling, amusements, and other subjects, all so thoughtfully considered that many customs are placed before us in a new light, and are given their true significance in our training of little minds and bodies. Volume VII. is now ready, and we feel that we shall earn the gratitude of every mother, who, following our advice, possesses herself of so excellent an addition to her library.

At the last meeting of the Hospitals Association at Guy's Hospital, Mr. G. A. Cross, Hon. Secretary of the Charities Rating Exemption Society, read a paper on the question, "Should Charities Pay Rates?" He pointed out that the work of hospitals and other public charities tended to the direct relief of the poor, and consequently of the poor rate, by preventing the class just above pauperism from becoming chargeable to the parish when overtaken by accident, affliction, or disease, and that in various ways they contributed to the health and welfare of the public at large. He contended that voluntary benevolence ought to be encouraged, and not discouraged, and remarked that if charitable institutions were taxed the supporters were subjected to a double burden, inasmuch as, besides contributing to charitable funds they paid the same rates as those who supported no charity at all. If viewed fairly, the money given for charitable purposes had already been taxed in the assessments of the givers. In the case of the parish of Chelsea, which had been called the "Village of Hospitals," the increase in the rates might amount to a little over ½d. in the pound were the exemption were to fall upon the ratepayers. In Ireland the whole of the public charities were free from rates and taxes, and in Scotland they were free, whilst in the Colonies they were not only exempt from taxation, but actually received large contributions from the public Treasury. He thought the simplest, shortest, and safest method of solving the question was by an appeal to both Legislative Houses in order that it might appear to the country whether Parliament really intended that the money given in bounty for the poor, the sick, and the afflicted, should suffer a serious reduction before it reached those for whom it was designed, and whether it desired that one-third of the United Kingdom should stand alone among the countries of the world in enduring a tax upon its own feelings of humanity and compassion.

A discussion followed the reading of Mr. Cross's paper before the Hospitals Association, and, on the motion of Dr. Steele, seconded by Mr. J. P. Stilwell, a resolution was unanimously adopted approving of the Bill drafted by the Charities Exemption Society for the purpose of restoring to charities the exemption from rates formerly enjoyed by them, and recommending the Chairmen and Treasurers of the leading London charities to further its passage through Parliament.

The Hampstead Home Hospital and Nursing Institute, N.W., is an institution which encourages thrift by helping those who are able and willing to help themselves. It was established in 1882, at 4, Parliament Hill Road, has steadily progressed, and now comprises five houses. The cost for alterations and furniture has been a heavy tax upon the resources of the Council, who greatly need some liberal donations to enable them to carry on their work. Nearly 200 patients were treated last year. The Hon. Secretary is Mr. R. A. Owthwaite, and the Sister Superintendent Mrs. Ebbetts.

